# Classifying and characterizing the development of self-reported overall quality of life among the Chinese elderly: a twelve-year longitudinal study

**DOI:** 10.1186/s12889-022-13314-6

**Published:** 2022-06-07

**Authors:** Xitong Huang, Minqiang Zhang, Junyan Fang, Qing Zeng, Jinqing Wang, Jia Li

**Affiliations:** 1 School of Psychology, South China Normal University, West of Zhongshan Avenue, Tianhe District, Guangzhou City, 510631 Guangdong Province China; 2 Key Laboratory of Brain, Cognition and Education Sciences (South China Normal University), Ministry of Education, Guangzhou, China; 3 Center for Studies of Psychological Application, South China Normal University, Guangzhou, 510631 China; 4 Guangdong Key Laboratory of Mental Health and Cognitive Science, South China Normal University, Guangzhou, 510631 China

**Keywords:** Chinese elderly, Chinese longitudinal healthy longevity survey, Self-reported overall quality of life, Growth mixture model

## Abstract

**Background:**

To promote healthy aging, the information about the development of quality of life (QoL) is of great importance. However, the explorations of the heterogeneity in the change of QoL under the Chinese context were limited. This study aimed to identify potential different development patterns of QoL and the influential factors using a longitudinal, nationally representative sample of the Chinese elderly.

**Methods:**

We adopted a five-wave longitudinal dataset from the Chinese Longitudinal Healthy Longevity Survey (CLHLS), and a total of 1645 elderly were obtained. The sample had a mean age of 72.7 years (SD = 6.64) and was 47.2% male. Overall QoL was measured through self-report during the longitudinal process. We utilized the conditional growth mixture model (GMM) with time-invariant covariates (TICs) to explore various development patterns and associated factors.

**Results:**

Three distinct trajectories of self-reported overall QoL were identified: the High-level Steady Group (17.08%), the Mid-level Steady Group (63.10%), and the Low-level Growth Group (19.82%). Results also indicated that several factors predicted distinct trajectories of self-reported overall QoL. Those elderly who received enough financial resources, had adequate nutrition, did not exhibit any disability, engaged in leisure activities, and did less physical labor or housework at the baseline were more likely to report a higher level of overall QoL over time.

**Conclusions:**

There existed three development patterns of self-reported overall QoL in elders, and the findings provided valuable implications for the maintenance and improvement of QoL among the Chinese elderly. Future studies could examine the influence of other confounding factors.

## Introduction

Population aging is occurring worldwide. It is predicted that the proportion of people aged 60 and above will be as high as 22% in the global population by 2050 [1, 2]. The accelerating of the aging process had aroused attention all over the world and the life situation of old people has sparked considerable scientific interest [1]. According to the Statistical Bulletin on National Economic and Social Development in 2018 [3], the proportion of elderly who aged above 60 reached 17.9% in the Chinese population, with the number at 249 million. Thus, the issue of healthy aging in China is worthy of attention [4–6].

One of the key factors that contribute to healthy aging is a high level of quality of life (QoL) [1, 7]. QoL is typically defined as an individual’s general perception of their position in life, which encompasses feelings of personal well-being, satisfaction with life, and self-worth [7, 8]. The academic interest in older people’s QoL has increased recently [7, 9]. QoL has been widely used as a health-related outcome in research about diseases, like dementia [10], cancer [11], and insomnia [12]. Besides, the level of QoL was found to be associated with various socio-demographic, health-related, and lifestyle factors, like smoking status [13], wealth status [8], physical status [10], and nutritional status [11].

To promote healthy aging, a full picture of QoL among the elderly is warranted. Up to now, the longitudinal explorations of QoL among the elderly have been largely limited to the developed world [8, 14–16], and only a few research focused on the Chinese population. Researchers identified five distinct change trajectories of QoL in a large and heterogeneous sample of older New Zealanders, which demonstrated that improving, maintaining, and declining QoL was possible to exist in later life simultaneously [17]. Evidence also suggested that there might exist considerable heterogeneity during the development of QoL in the Chinese old population [9], while the consensus about the classification results has not been reached. For example, scholars recognized a consistent tendency of increasing QoL among people with different cognitive statuses [18], while another study argued that the development of QoL might take on two kinds of trajectories by making an analogy to health well-being [19]. These inconsistent results warrant further exploration of the heterogeneity in the change of QoL under the Chinese context. Moreover, the exploration of influencing factors of QoL has been very limited in previous studies, thus, different protective or risk factors are also worthy of attention.

Considering that the classification approaches used in previous studies among the Chinese population were more in line with an artificial perspective or an analogical perspective, the present study intends to use a powerful analytic technique, the Growth Mixture Model (GMM) [20] to identify the heterogeneity during the change process. The main advantage of GMM is that it doesn’t rely on the assumption that all participants are drawn from a single population, which is the limitation of the traditional longitudinal model like the Latent Growth Model [21]. GMM aims to explain longitudinal heterogeneity through the identification of unobserved sub-populations in the sample under research [20, 22]. In GMM, longitudinal heterogeneity is captured by the inclusion of a categorical latent variable that identifies potential different development patterns, and the probabilities of classification for each individual are estimated to avoid the subjectivity of artificial grouping [20, 22, 23]. Furthermore, several covariates can be included in the GMM, which is called the conditional GMM, to identify the factors affecting these development patterns [22, 23].

The current study aims to identify potential distinct trajectories of QoL and the influential factors of the trajectory membership among the Chinese old population using the conditional GMM. To our knowledge, this is the first study to present the potential growth patterns of QoL and the influential factors under the Chinese context from the person-centered viewpoint. By identifying the underlying trajectories that had not been recognized before, our findings could advance the understanding of how QoL changes in different people, besides, exploring the influencing factors during the development process of QoL could provide insights for health researchers and policymakers so that early interventions can be taken to promote the QoL and improve the caring of the Chinese elderly. The following two questions will be addressed:

1. Are there potential distinct trajectories of QoL among the Chinese elderly individuals? What are the characteristics of these trajectories?
2. Which specific factors affect the trajectory membership? Are these effects positive or negative?

## Methods

### Study design and sample

The data we used were from the Chinese Longitudinal Healthy Longevity Survey (CLHLS), a collaborative effort between Duke University in the United States and Peking University in China. With the emphasis on the oldest-old from 22 provinces in mainland China, the CLHLS collected face-to-face interviews with the elderly in 1998, 2000, 2002, 2005, 2008, 2011, and 2014 [24]. The CLHLS interviewed all centenarians in the sampled provinces, and various sources whenever available were used to validate the accuracy of their age, including the birth certificate, genealogical documents, and household booklets [24]. The CLHLS collected data using internationally compatible questionnaires administered by trained investigators. All participants provided informed consent forms [24–26].

**Fig. 1:**
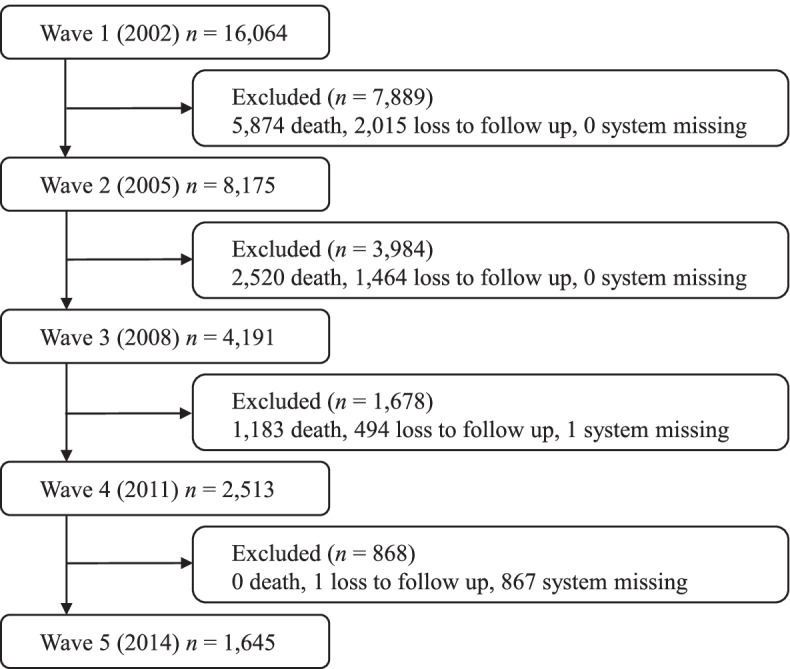
Structure of the analytic sample

For this study, we used the surveys from 2002 to 2014, which consisted of a five-wave dataset. The original sample of 16,064 individuals was interviewed in 2002 (T1), and participants were excluded if they died, lost to follow up, or failed to report on the index of self-reported overall QoL. Ultimately, a total of 1645 of the 2002 initial interviewees who were re-interviewed in 2014 were included. Figure 1 illustrated the structure of the analytic sample included in this study and the reasons for dropout in each measurement wave. The sample analyzed here had a similar sex ratio (47.2% male) to those participants who were excluded (42.1% male), and the proportions of Han ethnic were also very similar (92.8, 94.6%). Our sample was slightly younger (mean age at T1 was 72.7) than the excluded individuals (mean age at T1 was 87.9). The average scores of self-reported overall QoL at T1 were similar between our sample (3.67) and the excluded participants (3.66).

### Measures

#### Self-reported overall quality of life (QoL)

QoL is thought to encompass several aspects of life, like emotional functioning, cognitive functioning, social functioning, and spiritual well-being [27], but scholars agreed that a one-dimensional measure was adequate to represent it [28]. Measuring QoL with a single item has proven to be psychometrically sound to provide a global view of an individual’s QoL which refers to the overall QoL [27, 29]. The single-item scale of overall QoL has been widely used in empirical studies [30, 31]. Similar to previous studies, the CLHLS adopted a single item to measure overall QoL, which asked the participants to report their feeling about overall life quality with a Likert scale: “very good (1)”, “good (2)”, “so so (3)”, “bad (4)”, “very bad (5)”. To ensure a better understanding, this research reversely treats the score as “very bad (1)” to “very good (5)”, thus, a higher score represented a higher level of self-reported overall QoL.

#### Covariates

Several time-invariant covariates (TICs) were considered, which have been recognized as important to the elder’s QoL in previous studies [10, 11, 13]. All covariates were collected in 2002, the first wave of data collection, which included 6 basic variables (age, gender - “male = 1, female = 0”; ethnicity - “Han = 1, non-Han = 0”; financial source - “enough = 1, not enough = 0”; smoking status - “current smoker = 1, not current smoker = 0”; drinking status - “current drinker = 1, not current drinker = 0”), 5 dietary variables (eat fresh fruit, eat meat, eat fish, eat egg, drink tea), 6 functional variables (bathing disability, dressing disability, toileting disability, transferring disability, continence disability, feeding disability) and 5 behavioral variables (do physical labor regularly, do housework, read newspapers/books, watch TV or listen to the radio, take part in some social activities). All the dietary, functional, and behavioral variables were coded as 1 for “yes” and 0 for “never”.

### Analysis

Rates of missing data were generally 1.87% for self-reported overall QoL and 0.03% for TICs. Multiple imputations were used to handle missing data with five imputed datasets.

**Fig. 2:**
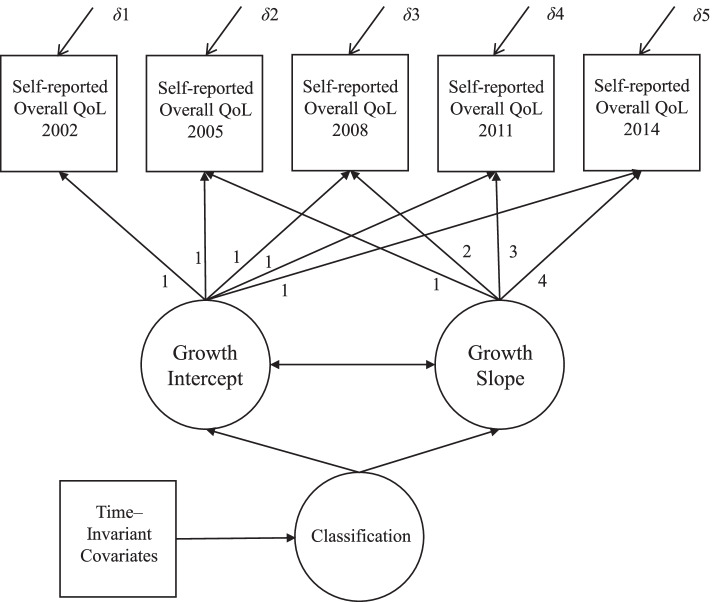
Structural model of the conditional GMM

Figure 2 presents the structural model of the conditional GMM using self-reported overall QoL as an illustration. All TICs mentioned above were included in the conditional GMM. In the analyses model, means of intercepts and slopes were allowed to vary between classes and within classes [32].

A series of GMMs of 1–4 classes were estimated to distinguish different trajectories of self-reported overall QoL among elderly individuals. Each *k*-class model was compared to a *k*-1 class model, and several indices were used to select the most optimal model, including the Akaike’s Information Criterion (AIC), Bayesian Information Criterion (BIC), Sample-size Adjusted Bayesian Information Criterion (SABIC), Entropy, Lo-Mendell-Rubin likelihood ratio test (Lo-Mendell-Rubin LRT), and Lo-Mendell-Rubin Adjusted Likelihood Ratio Test (Lo-Mendell-Rubin Adjusted LRT). Lower AIC, BIC, and SABIC values are indicative of better model fit. Significant LRT tests favor the *k* class model over the *k*-1 class model. Higher entropy indicates greater model fit. Once the optimal model was selected, TICs were entered into the model as predictors of the latent class membership (see Fig. 2).

Multiple imputations and descriptive statistics were calculated using SPSS 24.0 [33]. All GMMs were estimated using the Mplus 8.0 [34] with the Full-Information Maximum Likelihood (FIML) estimation.

## Results

### Descriptive statistics

Descriptive statistics for the self-reported overall QoL measured from 2002 to 2014 are presented in Table 1. Over the 12 years, the average scores of self-reported overall QoL gradually increased from 3.67 (*SD* = 0.81) to 3.80 (*SD* = 0.83).

**Table 1:** Descriptive statistics for the analyzed variables

Analyzed Variables	Mean (*SD*)
Focal variables	
self-reported overall QoL (2002)	3.67(0.81)
self-reported overall QoL (2005)	3.67(0.81)
self-reported overall QoL (2008)	3.68(0.80)
self-reported overall QoL (2011)	3.74(0.87)
self-reported overall QoL (2014)	3.80(0.83)
Basic variables (2002)	
Age	72.73(6.64)
Gender, male	0.47(0.50)
Ethnicity, Han	0.93(0.26)
Financial resources, enough	0.81(0.39)
Smoking status, current smoker	0.25(0.43)
Drinking status, current drinker	0.25(0.43)
Dietary variables (2002)	
Eat fresh fruit	0.79(0.41)
Eat meat	0.85(0.36)
Eat fish	0.74(0.44)
Eat eggs	0.86(0.35)
Drink tea	0.53(0.50)
Functional variables (2002)	
Bathing disability	0.03(0.17)
Dressing disability	0.01(0.09)
Toileting disability	0.01(0.08)
Transferring disability	0.00(0.07)
Continence disability	0.00(0.06)
Feeding disability	0.00(0.07)
Behavioral variables (2002)	
Do physical labor regularly	0.85(0.35)
Do housework	0.87(0.34)
Read newspapers/books	0.27(0.45)
Watch TV or listen to the radio	0.84(0.37)
Take part in some social activities	0.21(0.41)

Table 1 also presents the descriptive statistics of all the 22 covariates at the baseline. As can be seen, the mean age of the elderly individuals was 72.73 (*SD* = 6.64), and more than half of them were female. Most of the elderly were of Han ethnic background and reported to have enough financial resources. Nearly one-quarter of the elderly did not smoke or drink alcohol at the baseline. Meat and eggs were the most favorite food, and they were eaten by more than 80% of the elderly participants. Almost all the elderly did not exhibit any functional disability. The elderly individuals who reported doing physical labor regularly took up more than 80% of the sample, the same for the elderly who did housework, watched TV, or listened to the radio during leisure time.

### Conditional GMM with covariates

#### Fitting result

Table 2 presents the fitting results of several models. As can be seen, the AIC, BIC, and SABIC had no agreement on which model fitted better. The entropy value of the 3-class solution was the largest among the solutions, which meant that the best solution was probably the 3-class solution. Additionally, both the Lo-Mendell-Rubin LRT and Lo-Mendell-Rubin Adjusted LRT showed that the 2-class solution fitted better than the 1-class solution (*p* < 0.001), the 3-class solution fitted better than the 2-class solution (*p* < 0.05) and the 4-class solution fitted better than the 3-class solution (*p* < 0.001). This suggested that the best solution contained at least 4 classes. However, the interpretability of the 4-class solution was limited because two of the four classes had a similar change pattern. For the sake of parsimony, we chose the 3-class solution finally after considering the performance of all indicators comprehensively.

**Table 2:** Fitted indices for GMMs with 1 to 4 classes

Number of Classes	AIC	BIC	SABIC	Entropy	Lo-Mendell-Rubin LRT	Lo-Mendell-Rubin Adjusted LRT
1	19,643.55	19,697.61	19,665.84	–	–	–
2	19,324.69	19,513.88	19,402.69	0.65	vs 1 35.81^***^	vs 1 366.88^***^
3	19,238.99	19,563.32	19,372.71	0.89	vs 2 246.73^*^	vs 2 223.99^*^
4	19,235.89	19,695.36	19,425.33	0.85	vs 3 49.01^***^	vs 3 48.82^***^

*AIC* Akaike’s Information Criterion, *BIC* Bayesian Information Criterion, *SABIC* Sample-size Adjusted Bayesian Information Criterion, *Lo-Mendell-Rubin LRT* Lo-Mendell-Rubin likelihood ratio test, *Lo-Mendell-Rubin Adjusted LRT* Lo-Mendell-Rubin Adjusted Likelihood Ratio Test

^*^p < 0.05

^**^p < 0.01

^***^p < 0.001

#### Three-class GMM

**Fig. 3:**
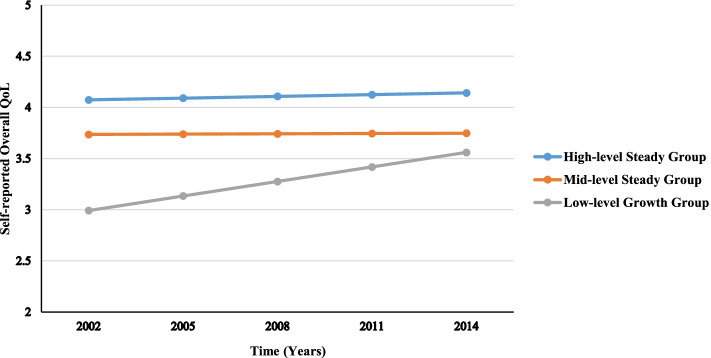
Development trends of self-reported overall QoL

Figure 3 presents the development trends of self-reported overall QoL in the 3-class solution. The total number of participants in the first group was 281, which accounted for 17.08% of the sample. The first group had a high initial level and a steady trend of self-reported overall QoL, with the mean of the intercept being 4.07 (*SE* = 0.04, *p* < 0.001) and the mean of the linear slope being 0.02 (*SE* = 0.01, *p* > 0.05). Therefore, this group was labeled as the High-level Steady Group.

The second group (*n* = 1038) which accounted for 63.10% of the sample showed a medium initial level and a steady trend. The mean of intercept was 3.74 (*SE* = 0.11, *p* < 0.001) and the mean of the linear slope was 0.00 (*SE* = 0.02, *p* > 0.05), therefore, this group was named the Mid-level Steady Group.

The third group (*n* = 326) accounted for 19.82% of the sample, and it presented a low initial level and an increasing trend. In the third group, the mean of intercept was 2.99 (*SE* = 0.05, *p* < 0.001) and the mean of the linear slope was 0.14 (*SE* = 0.03, *p* < 0.001), therefore, this group was labeled as the Low-level Growth Group.

#### The impact of covariates

After identifying the development trends of self-reported overall QoL, influential factors of these different trajectories were examined. In this study, we considered 6 basic variables, 5 dietary variables, 6 functional variables, and 5 behavioral variables. These covariates were included in the GMM as TICs, which were all collected at the baseline (in 2002). The Low-level Growth Group was used as the reference group. Table 3 presents the effects of these covariates in the 3-class solution.

**Table 3:** The impact of covariates on the development trends of self-reported overall QoL in the 3-class solution

Covariates	High-level Steady Group		Mid-level Steady Group	
	OR	95% CI	OR	95% CI
Reference: the Low-level Growth Group				
Basic variables (2002)				
Age	1.02	(0.99–1.05)	0.99	(0.97–1.01)
Gender, male	2.84^***^	(2.02–3.98)	0.73	(0.57–1.94)
Ethnicity, Han	3.21^**^	(1.44–7.16)	1.10	(0.70–1.72)
Financial resources, enough	1103.03^***^	(152.00–8004.62)	81.25^***^	(54.68–120.74)
Smoking status, current smoker	0.78	(0.55–1.12)	0.63	(0.48–1.44)
Drinking status, current drinker	1.52	(1.07–2.17)	0.97	(0.72–1.29)
Dietary variables (2002)				
Eat fresh fruit	23.31^***^	(12.82–42.37)	6.30^***^	(4.78–8.30)
Eat meat	4.44^***^	(2.67–7.38)	2.16^***^	(1.59–2.91)
Eat fish	6.02^***^	(3.80–9.55)	1.69^***^	(1.30–2.19)
Eat eggs	2.83^***^	(1.75–4.57)	1.91^***^	(1.39–2.63)
Drink tea	2.20^***^	(1.59–3.05)	1.68^***^	(1.30–2.16)
Functional variables (2002)				
Bathing disability	1.98	(0.48–2.65)	1.10	(0.36–3.37)
Dressing disability	0.33	(0.07–1.59)	0.13^**^	(0.03–0.51)
Toileting disability	0.77	(0.13–4.65)	0.52	(0.12–2.19)
Transferring disability	0.58	(0.11–3.17)	0.08^*^	(0.01–0.70)
Continence disability	–	–	–	–
Feeding disability	0.77	(0.13–4.65)	0.21	(0.04–1.25)
Behavioral variables (2002)				
Do physical labor regularly	0.12^***^	(0.07–0.19)	0.71	(0.45–1.12)
Do housework	0.23^***^	(0.15–0.36)	1.09	(0.72–1.67)
Read newspapers/books	23.05^***^	(14.97–35.50)	1.69^**^	(1.17–2.45)
Watch TV or listen to the radio	11.17^***^	(5.51–22.68)	1.96^***^	(1.46–2.63)
Take part in some social activities	25.29^***^	(15.97–40.08)	1.28	(0.83–1.96)

All the elderly did not exhibit continence disability

*OR* odds ratio, *95% CI* 95% Confidence Interval

^*^p < 0.05

^**^p < 0.01

^***^p < 0.001

It was found that men had higher odds of reporting a high level of overall QoL over time than women (OR = 2.84, 95% CI: 2.02–3.98, *p* < 0.001 for the High-level Steady Group), those who had Han ethnic background were more likely to report a high level of overall QoL constantly (OR = 3.21, 95% CI: 1.44–7.16, *p* < 0.01 for the High-level Steady Group), and the odds of reporting a higher level of overall QoL over time were significantly higher for those who received enough financial sources (OR = 1103.03, 95% CI: 152.00–8004.62, *p* < 0.001 for the High-level Steady Group; OR = 81.25, 95% CI: 54.68–120.74, *p* < 0.001 for the Mid-level Steady Group).

Concerning the dietary factors, our results showed that those who ate fresh fruit were related to higher odds of reporting a higher level of overall QoL over time (OR = 23.31, 95% CI: 12.82–42.37, *p* < 0.001 for the High-level Steady Group; OR = 6.30, 95% CI: 4.78–8.30, *p* < 0.001 for the Mid-level Steady Group). And same was true for those who ate meat (OR = 4.44, 95% CI: 2.67–7.38, *p* < 0.001 for the High-level Steady Group; OR = 2.16, 95% CI: 1.59–2.91, *p* < 0.001 for the Mid-level Steady Group), fish (OR = 6.02, 95% CI: 3.80–9.55, *p* < 0.001 for the High-level Steady Group; OR = 1.69, 95% CI: 1.30–2.19, *p* < 0.001 for the Mid-level Steady Group), eggs (OR = 2.83, 95% CI: 1.75–4.57, *p* < 0.001 for the High-level Steady Group; OR = 1.91, 95% CI: 1.39–2.63, *p* < 0.001 for the Mid-level Steady Group), and those who drank tea (OR = 2.20, 95% CI: 1.59–3.05, *p* < 0.001 for the High-level Steady Group; OR = 1.68, 95% CI: 1.30–2.16, *p* < 0.001 for the Mid-level Steady Group).

Regarding the functional disability factors, the results indicated that those who exhibited dressing disability were more likely to report a lower level of overall QoL over time (OR = 0.13, 95% CI: 0.03–0.51, *p* < 0.01 for the Mid-level Steady Group), and same for those had exhibited transferring disability (OR = 0.08, 95% CI: 0.01–0.70, *p* < 0.05 for the Mid-level Steady Group).

As for the behavioral factors, those who did physical labor regularly were more likely to report a lower level of overall QoL over time (OR = 0.12, 95% CI: 0.07–0.19, *p* < 0.01 for the High-level Steady Group), same for those who did housework (OR = 0.23, 95% CI: 0.15–0.36, *p* < 0.001 for the High-level Steady Group). Furthermore, the odds of report a higher level of overall QoL over time were significantly higher for those who read newspapers or books (OR = 23.05, 95% CI: 14.97–35.50, *p* < 0.001 for the High-level Steady Group; OR = 1.69, 95% CI: 1.17–2.45, *p* < 0.01 for the Mid-level Steady Group), those who watch TV or listen to the radio (OR = 11.17, 95% CI: 5.51–12.68, *p* < 0.01 for the High-level Steady Group; OR = 1.96, 95% CI: 1.46–2.63, *p* < 0.001 for the Mid-level Steady Group), and those who took part in some social activities (OR = 25.29, 95% CI: 15.97–40.08, *p* < 0.001 for the High-level Steady Group).

## Discussion

Based on the five-wave longitudinal data from a large representative Chinese sample, the present study examined the variability in the development of self-reported overall QoL among the elderly. The results showed that subjects could be categorized into three groups based on how their self-reported overall QoL changed, which provided empirical evidence for the supposition that there existed heterogeneity in the development of QoL among the elderly [1, 7, 9].

The first group was the High-level Steady Group which consisted of 17.08% of the sample. Their average self-reported overall QoL ratings were higher than the other two groups and changed very little over 12 years. This was consistent with previous literature which reported that there were a certain number of old people who reported excellent QoL [1, 35]. The second group was the Mid-level Steady Group, and it took up the highest proportion (63.10%). Our findings showed that the self-reported overall QoL of the elderly in this group stayed at a stable and relatively high level. Thus, it could be inferred that more than half of the elderly held a rather positive attitude towards their life. The third group, which was called the Low-level Growth Group, accounted for 19.82% of the sample. The self-reported overall QoL in this group showed a low initial level and an increasing trend. Notably, the overall QoL level in this group was constantly lower than the other two groups, and this subgroup of elderly who kept reporting poorer QoL had been reported in previous research [17]. This classification result was similar to a previous study, which reported three classes of QoL perception among Hong Kong residents using a classification tree approach [36]. However, their findings were based on cross-section data so development information was lost. The present study focused on the change of QoL by analyzing a large longitudinal dataset, and we revealed the subgroup heterogeneity during the dynamic development. Collectively, our results indicated that most elderly in China held a positive attitude to their life with an increasing or stable perception of overall QoL, while there still existed a few reporting poorer QoL over time. Furthermore, although these few elderly reported that their perception of overall QoL was increasing as time went by, it had always been lower than the others over time. Besides, it was found that frail older people with a lower level of QoL reported more unmet needs [1]. Then the possible reasons for this phenomenon were discussed below.

Our findings showed that several protective or risk factors were linked to the perception of overall QoL, which could help promote the understanding of QoL among elders in China. First, the results indicated that old people with enough financial sources were more likely to be grouped to the High-level Steady Group or Mid-level Steady Group rather than the Low-level Growth Group. This could be interpreted as follows. The elderly with greater financial resources could afford medical products to get timely treatment when needed [37], and they were able to enjoy better social services that could help them overcome difficulties encountered in daily life [38]. In addition, people with greater financial resources were less likely to suffer from financial strain that was related to poorer living environments, diet, and other daily necessities [39]. Enough financial resources could also enable the elderly to be more optimistic, have more positive perceptions and expectations about their future, and feel more useful to others [6]. Overall, our results were consistent with the previous research which found that the elderly who had better financial resources were more likely to report a higher level of QoL over time [8].

Second, our results underscored the importance of adequate nutrition for the elderly. In the present study, higher perceptions of overall QoL were associated with consuming fresh fruit, meat, fish, eggs, and tea. Fresh fruit consisted of carotenoids, and it could benefit muscle strength [40], protect against coronary heart disease [41], and prevent negative health outcomes like obesity, hypertension, type 2 diabetes, and vascular disease [42]. The meat contained several vitamins and minerals, as well as all essential amino acids, making it an excellent protein source for building and maintaining muscle. In alignment with this, meat constituted an important part of the diet for the elderly to prevent age-related declines in muscle strength and frailty (sarcopenia) [43]. Fish was low in saturated fat and high in protein. The ω-3 essential fatty acids derived from fish—eicosapentaenoic acid and docosahexaenoic acid—had been shown to decrease inflammation and be useful in depression, Alzheimer’s disease, and rheumatoid arthritis [44]. Eggs also were an important source of protein and other valuable nutrients. The components of eggs, phospholipids, vitamin E, and folic acid were extremely important in preventing the development of atherosclerosis and other metabolic syndromes. Besides, it was also worth mentioning that lutein and zeaxanthin could form a protective barrier against the degeneration of the macula of the human eye [45]. As one of the most consumed beverages in the world [46], tea consisted of theaflavins, catechins, metabolites, and polyphenols, which could be efficient for the prevention and treatment of numerous metabolic disorders [47]. In short, adequate nutrition might make the elderly healthier and thus lead to higher QoL. Nutritional screenings and interventions for elders at risk for malnutrition would improve their life quality to our knowledge about the dietary needs of China’s elders, who grew up in a material-deprived society without much calcium or protein in their diet [19].

Third, it was found that the elderly with dressing disabilities or transferring disabilities were more likely to be grouped to the Low-level Growth Group rather than the Mid-level Steady Group. This result was consistent with previous findings which found that lower perception of QoL was associated with more serious functional disability among the elderly [48]. One possible explanation was that disabled older adults’ self-care ability deteriorated and then their normal physiological activities were restricted. Such a process affected their social interaction and mental health adversely, and this reduced their perception of QoL ultimately [49]. Besides, physical health was a strong predictor of psychological well-being which was closely connected with the perception of QoL among the elderly [50].

Fourth, our findings provided evidence of the importance of engaging in leisure activities [42]. In the current study, several activities were related to better subject QoL, like reading newspapers or books, watching TV or listening to the radio, and taking part in some social activities. Leisure activities had been regarded as protective factors for QoL perception because they could enhance motivation, and provide social support and meaning in life [42]. Besides, our results also revealed that doing physical labor and housework affected overall QoL negatively. This was consistent with previous studies. For example, it was found that washing clothes and house cleaning were negatively linked to older adults’ health [51]. It meant that doing physical labor and housework could be burdensome for the elderly, which then reduced their perception of QoL. Furthermore, it was worth noting that, women were more likely than men to report poorer QoL in the current study, and this could be ascribed to the gendered work-life imbalance [52], which had been confirmed by previous studies [51–54].

Overall, the present results indicated that those elderly who received enough financial resources and adequate nutrition did not exhibit any disability, engaged in leisure activities, and did less physical labor or housework were more likely to report a higher level of overall QoL about aging. These findings provided valuable implications for the caring of the Chinese elderly. For example, welfare institutions must be established to provide sufficient living resources to those who had financial difficulties. Efficient interventions and programs should be implemented to prevent disability among the elderly. In addition, society and families should encourage the elderly to engage in leisure activities.

The strengths of the study included: 1) we analyzed longitudinal data from the CLHLS, which was a large multi-wave nationally representative longitudinal study concerning the general older population in China, and this ensured the generalizability of our findings. 2) We employed a novel analyses method, the conditional GMM, to identify the potential development trends of self-reported overall QoL, and this was able to avoid limitations in the traditional artificial grouping. 3) Our findings examined a range of covariates, and this went beyond previous studies by being able to recognize important influential factors of the development of self-reported overall QoL. Previous evidence about the covariate effects on QoL was mostly from cross-sectional surveys [10, 11, 13], while our study investigated the covariate effects from a longitudinal perspective. These findings could help promote the understanding of different covariate effects and provide valuable information for practice. However, this study also had some shortcomings. First, several previous studies reported that elderly women spent more time on housework activities and typically performed more routine and repetitive tasks than men [51–54], and this might be one of the reasons why women were more likely to report poorer QoL than men [53]. It would be interesting to explore various change trends of QoL in different gender groups separately. Second, the findings need to be used with caution because of the potential bias that might be induced by attrition which is almost inevitable in longitudinal studies with a long timeline. Third, the results may be limited since using self-reported overall QoL is not a good substitute for well-known and standard health-related QoL measures. Additionally, this study only included time-invariant covariates, and future studies could consider some time-varying covariates.

## Conclusions

This study used the conditional GMM to examine various development trajectories of self-reported overall QoL and the influential factors in a nationally representative Chinese sample across 12 years. Three subgroups with distinct trajectories were identified: the High-level Steady Group, the Mid-level Steady Group, and the Low-level Growth Group. Furthermore, the findings suggested that the elderly who received enough financial resources and adequate nutrition did not exhibit any disability, engaged in leisure activities, and did less physical labor or housework were more likely to report a higher level of overall QoL over time. These findings helped promote the understanding of QoL among the Chinese elderly and provided valuable implications for the practice of elderly care.

## Data Availability

The CLHLS datasets are publicly available at the National Archive of Computerized Data on Aging (http://www.icpsr.umich.edu/icpsrweb/NACDA/studies/03891). Researchers may obtain the datasets after submitting a data user agreement to the National Archive of Computerized Data on Aging.

## Abbreviations

QoL: Quality of Life
CLHLS: Chinese Longitudinal Healthy Longevity Survey
GMM: Growth mixture model
